# Anti-Icing and Frost Property of Superhydrophobic Micro-Nano Structures with Embossed Micro-Array Channels

**DOI:** 10.3390/ma18204813

**Published:** 2025-10-21

**Authors:** Han Luo, Xiaoliang Wang, Qiwei Li, Honglei Liu, Lei Chen, Debin Shan, Bin Guo, Jie Xu

**Affiliations:** 1 School of Materials Science and Engineering, Harbin Institute of Technology, Harbin 150001, China; 25b309045@stu.hit.edu.cn (H.L.); shandb@hit.edu.cn (D.S.); guobin@hit.edu.cn (B.G.); 2 National Key Laboratory for Precision Hot Forming, Harbin Institute of Technology, Harbin 150001, China; liqiwi@163.com; 3 Northeast Light Alloy Co., Ltd., Harbin 150060, China; hl77238@163.com

**Keywords:** anti-icing, superhydrophobic, aluminum alloy, hot embossing, microarray channels

## Abstract

Icing on aircraft surfaces during operation poses a threat to flight safety. As a passive anti-icing technology, hydrophobic microstructure can achieve long-term anti-icing. In this work, a composite process combining hot-embossing of PVD-coated punches with a low surface energy fluoride-modification scheme is proposed to generate nanoscale cluster structures on hundreds of microns array channels to construct a superhydrophobic micro-nano composite structure. The droplet freezing and frosting behavior of the hydrophobic microstructures was analyzed, and it was found that the anti-icing and anti-frost properties of the microstructure surface improved with an increase in the microstructure period size (*T*). Compared with the original surface, the freezing time of the microstructure at *T* = 500 μm was delayed by 214.3% (7 s → 22 s), and the frost layer coverage time was delayed by 75.7% (70 s → 123 s). The maximum water contact angle of the superhydrophobic micro-nano composite structure was 153.3°, and the droplet freezing time was delayed to 95 s, which is a 1166.67% difference, indicating that the multi-stage micro-nano composite structure can significantly improve surface anti-icing performance. The main reason for this result is that the bottom of the microstructure can store air pockets, preventing droplet wetting and heat exchange.

## 1. Introduction

Hydrophobic surface microstructures have functional properties such as anti-icing [1], drag reduction [2], antibacterial [3], and oil resistance [4] and have shown great application potential in aerospace [5], energy and power [6], biomedicine [7], marine vessels [8], and efficient thermal management [9]. However, when aircraft fly through clouds, supercooled water droplets or water vapor may cause ice to form or even accumulate on the surface of the aircraft, which poses a great challenge to flight safety and operational efficiency. Therefore, developing a long-term, highly effective aircraft anti-icing technology is of great importance.

The anti-icing capacity of hydrophobic microstructures originates from their distinctive surface geometry and chemical properties, a mechanism well-substantiated by the Cassie–Baxter model. This model illustrates that the microstructure can reduce heat exchange and nucleation sites by forming “air pockets” between the droplet and the microstructure [10], delay the freezing temperature of the supercooled droplet and slow down the crystallization process [11], and reduce the adhesion of the ice layer [12], thereby achieving surface anti-icing. Wang et al. [13] etched a microstructure on the surface of an aviation aluminum alloy, combined with subsequent chemical modification to obtain a nanoscale super-hydrophobic surface, and tested its anti-icing property in an artificial climate laboratory simulating a high-altitude atmospheric environment. It was found that the accumulated mass of the ice layer on the structure was much smaller than that on an ordinary surface. Jia et al. [14] used femtosecond laser-induced silicone rubber to prepare a superhydrophobic array microstructure with a water contact angle of 168° and a sliding angle of 0.3°. They wrapped the structure around the leading edge of a propeller and found that the dynamic anti-icing duration of the structure in an ice wind tunnel was 83.3% higher than that of a smooth surface. P. Hauschwitz et al. [15] prepared a super-hydrophobic microstructure surface with a water contact angle of up to 173° on the 7075 aluminum alloy commonly used in fuselages, which has excellent durability in atmospheric environments. Michael et al. [16] used ultrashort laser pulses to treat a 316 stainless steel substrate and combined it with a hydrothermal method to obtain a superhydrophobic surface. An ice wind tunnel was used to verify that the surface had the dual function of significantly improving the prevention of water adhesion and ice adhesion. These studies demonstrate that hydrophobic microstructures can reduce ice accumulation, delay icing, and maintain excellent stability in simulated high-altitude atmospheric environments, potentially solving the problem of anti-icing aircraft surfaces. However, hydrophobic microstructures are susceptible to wear and high-pressure impact, leading to structural damage and degradation of anti-icing property. Furthermore, the stability of the Cassie state of droplets on the microstructured surface needs to be further improved to achieve long-term, stable anti-icing. In the meantime, existing preparation methods present significant challenges in precisely controlling micro- and nanostructures, hindering the optimization of anti-icing properties.

Surface microstructures typically have feature sizes ranging from submillimeter to micro-nanometer. Issues with processing efficiency, precision, structural scale, and stability make their manufacturing significantly more challenging than traditional structural processing. Creating highly stable and precise surface microstructures has become a key technological bottleneck in achieving breakthroughs in aircraft surface anti-icing technology. Microstructure manufacturing technologies mainly include laser micromachining, micromachining, plastic microforming, etc. Wang et al. [17] used laser etching to construct a grid microarray structure on electrothermal materials and combined it with fluorinated silica modification to obtain superhydrophobicity, achieving low-power, long-lasting anti-icing and rapid ice melting at −30 °C. Li et al. [18] combined nanosecond laser ablation and hot stamping technology to prepare a T-shaped array microstructure on a pure copper surface. The structure has excellent anti-icing properties and a stable water contact state. After the ice melting cycle, the delayed freezing time at −17 °C exceeds 600 min. Zhou et al. [19] used ultraviolet laser irradiation and deposition technology to prepare a mosquito-eye structure on a pure copper surface. Its freezing delay time is 3 times that of the original plane, and the surface ice shedding time under outdoor conditions is only 49.92% of the original plane. Yu et al. [20] formed a microgroove array on the surface of an aluminum alloy by micro-milling and grinding. Jia et al. [21] used micro-milling to form a micro-pillar array structure on the surface of a Cu substrate and successfully prepared a superhydrophobic surface with excellent mechanical durability and corrosion resistance. Inspired by the microstructures of lotus leaf and cicada wing arrays, Liang et al. [22] successfully constructed an array microstructure on the surface of 7075 aluminum alloy via high-speed wire electric discharge cutting (HS-WEDM). This structure had a high apparent contact angle of 153° without chemical treatment, and the contact angle hysteresis was less than 5°, showing good superhydrophobic properties. Hou et al. [23] used plasma etching technology to construct a microcube array with a minimum pitch of 30 μm on the silicon surface. The surface contact angle could reach up to 154°, and the freezing time was delayed to 1295 s, which was two orders of magnitude lower than the initial plane. In addition, Zhang et al. [24] prepared a mechanically durable superhydrophobic coating via simple hot pressing sintering. Zgaren et al. [25] used LCD 3D-printing technology to manufacture superhydrophobic and low ice adhesion anti-icing surfaces. The ice adhesion of the prismatic array microstructure surface was only 18.89% of that of the flat surface. Yang et al. [26] prepared three hierarchical superhydrophobic microstructures on a Cu surface via pulse electrodeposition and verified through wear cycles that the surface with larger microstructures can better maintain superhydrophobicity under mechanical damage. Anti-icing surfaces can be manufactured through special processing methods such as laser etching, machining, and 3D printing. However, the low efficiency and high cost of the above forming methods hinder the further popularization and application of microstructured anti-icing surfaces.

Plastic microforming is a technology that uses plastic deformation to manufacture micro parts or microstructures. It has the advantages of a simple process, high efficiency, low cost, high precision, and superior mechanical properties. It is expected to achieve mass production of surface anti-icing microstructures.

Wang et al. [27] prepared a four-level pyramid array microstructure with full filling and a clear outline on aluminum alloy using micro-embossing technology, combined with the hydrothermal method, to construct a multi-level micro-nanostructure to achieve surface hydrophobic modification. They carried out droplet impact experiments and found that the droplets on the surface of the super-hydrophobic structure bounced completely. Srivastava et al. [28] used hot-embossing technology to manufacture array micropores and micro-column structures on the surface of 5083 aluminum alloy and used silicone oil to achieve surface hydrophobic modification. Combined with droplet impact experiments, it was found that the hydrophobic microstructure can still maintain its super-slip properties after being immersed in saltwater for 7 days. Xu et al. [29] used micro-molding technology to prepare microcube and microchannel array structures on the surface of pure copper and achieved super-hydrophobic modification through plasma treatment.

However, more studies have shown that hierarchical micro/nano-structures can significantly improve hydrophobicity compared to single microstructures. Shen et al. [30] observed frosting and defrosting processes on unmodified aluminum substrates, modified microstructures, modified nanostructures, and modified hierarchical micro/nanostructures and found that the frost layer and meltwater on the surface of micro-nano composite structures can spontaneously fall off faster. Zhu et al. [31] used a micro-milling and chemical modification composite process to manufacture a multi-level micro/nano surface with triangular grooves on an aluminum alloy substrate. Compared with the microstructure surface after micro-milling, the surface contact angle after hydrophobic modification increased by 18% at most and showed good corrosion resistance. Wang et al. [32] combined hot stamping with a nanosecond laser to construct a micro-nano composite structure through subsequent chemical modification and realized the transformation from a superhydrophilic wetting state to a superhydrophobic state on the surface of the microstructure. Compared with the V-shaped channel obtained via hot stamping, the hydrophobicity of the multi-stage composite structure was improved, and it tended to be isotropic and make the droplets slide down more easily. Xu et al. [33] prepared an array of micro-hexagonal columns with a characteristic size of 75 μm on ultrafine-grained pure aluminum via a micro-molding process and combined it with subsequent hydrophobic modification with stearic acid to form and fabricate superhydrophobic micro-nano composite structures. The maximum water contact angle of this micro-nano hierarchical structure is ~160°, which is 21.7% higher than that of a single microstructure.

Table 1 shows a comparison of some studies on microstructure anti-icing. It can be seen that the surface microstructure can improve the contact angle, delay icing, and reduce ice accumulation. At the same time, there is relatively little research on micro-mechanical formation technology on anti-icing surfaces, and most literature only characterizes the contact angle. There is little research on anti-icing performance.

Despite significant progress in hydrophobic microstructures for aircraft deicing, three critical gaps remain: Firstly, trade-off between structural stability and long-term deicing performance—existing microstructures exhibit short-term de-icing efficacy but are prone to damage from airflow erosion and abrasive impacts during flight, with mechanical durability failing to meet long-term service requirements. Secondly, limitations of single-microstructure hydrophobicity—individual micron- or nanoscale structures exhibit limited hydrophobic performance and poor Cassie state stability, readily transitioning to the Wenzel state upon droplet impact, leading to de-icing failure. Thirdly, large-scale production is difficult—while techniques like laser micro-machining and plasma etching enable high-precision microstructure fabrication, they suffer from low processing efficiency and high costs [17,23]. Traditional plastic-forming methods, such as thermal embossing, allow for mass production but struggle to create complex morphologies and require subsequent modification, resulting in limited improvements in hydrophobicity [27,29]. Based on this, the following work was conducted in this study:

(1) Triangular microarray channels were formed using a multi-arc ion punch-assisted thermal embossing process.
(2) The freezing delay behavior of individual droplets on microarray channels was investigated, along with the mechanisms governing surface frost formation and thawing.
(3) Micron- and nanoscale composite structures were constructed on microarray channels via low-surface-energy chemical modification, the anti-icing performance of these composite structures was validated, and the mechanism underlying microstructure-induced freezing delay was further elucidated.

## 2. Materials and Methods

### 2.1. Microarray Structure Embossing and Modification Scheme

**Figure 1:**
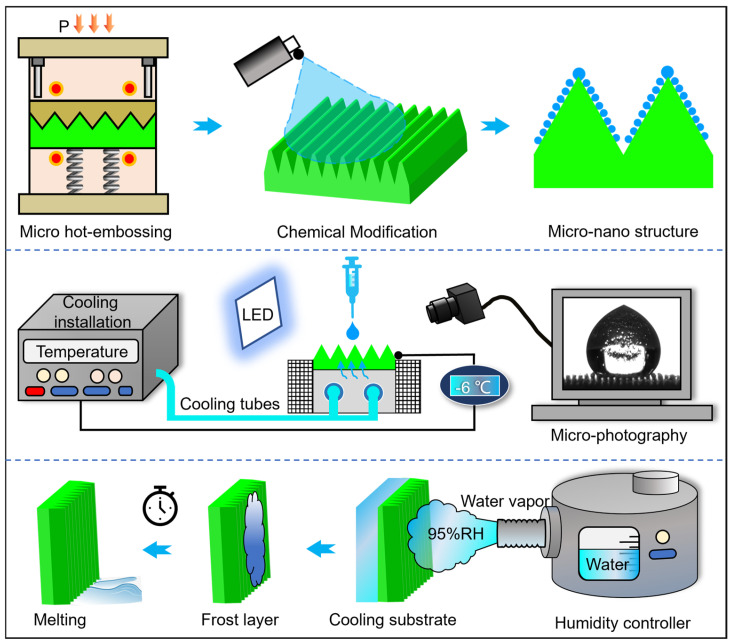
Microstructure formation, hydrophobic modification scheme, and property characterization.

A commonly used 7075 aluminum alloy for aeronautics was hot-embossed using a PVD (Physical Vapor Deposition) punch (Dalian V-Tech Nano Technologies Inc., Dalian, China) with Cr-Zr-N coating, creating triangular microarray channels with periodicities of 100 μm, 300 μm, and 500 μm, and a 45° angle at the top of the microstructure. To further enhance hydrophobicity, the surface was modified using a fluorinated silica solution. To further enhance hydrophobicity, the surface was modified using a fluorinated silica solution (15% fluorosilane polymer concentration, Shenzhen Weijing Advanced Materials Technology Co., Ltd., Shenzhen, China). As shown in Figure 1, the Systence XT1836-HEB-DX100 (Shanghai Xiteng Electronic Information Technology Co., Ltd., Shanghai, China) precision microforming system with a maximum load of 100 kN and a positioning accuracy of ±1 μm was used to press the microarray channels onto a 10 mm × 10 mm × 1 mm aluminum alloy surface using a PVD-coated punch. Formation parameters were based on previous work [34]: hot-embossing temperature of 350 °C, formation speed of 0.005 mm/s, and a holding pressure of 300 s before demolding. Fluoride has extremely low surface energy and is a commonly used hydrophobic modification agent. The embossed sample was ultrasonically cleaned in ethanol for 5 min and then dried. In a fume hood, a fluorosilane-modified nano-silica solution was evenly sprayed on the surface of the microchannel array. It was then kept in a preheated tube furnace for 10 min at 100 °C to generate a super-hydrophobic micro-nanostructure on the microstructure.

### 2.2. Microstructure Characterization Methods

The surface of the embossed microarray channels and the morphology of the hydrophobically modified nanostructures were observed using a TESCAN MAGNA (TESCAN GROUP, Brno, Czech Republic) high-resolution emission scanning electron microscope (SEM), and the three-dimensional morphology was measured using an Olympus laser confocal microscope (OLS-5000, OLYMPUS, Tokyo, Japan). A self-made freezing–frosting experimental platform was used to analyze the freezing behavior of droplets on the surface of microarray channels, as shown in Figure 1, including a circulating water-cooling system and a temperature control system (Shaoyang Yuliang Electronic Commerce Co., Ltd., Shaoyang, China). A microinjector was used to ensure that 5 μL of deionized water naturally drips on the semiconductor refrigeration platform each time. The circulating water-cooling system stabilizes the sample temperature at −6 °C and applies thermal grease between the sample and the cooling platform. In the blooming experiment, the fogger (LY-A1 intelligent humidifier, Dongguan Fenggang Laiyong Electrical Appliance Co., Ltd., Dongguan, China) can control the water vapor humidity at 95% and the temperature at 20 °C. The freezing and thawing processes were recorded by a contact angle measuring instrument (JC2000D2, Shanghai Zhongchen Digital Technology Equipment Co., Ltd., Shanghai, China). The water contact angle was measured by repeating the measurement three times using a contact angle measuring instrument (JC2000D2) and calculating the mean value and standard deviation.

## 3. Results and Discussion

### 3.1. Surface Characteristics of Microarray Channel Structures

**Figure 2:**
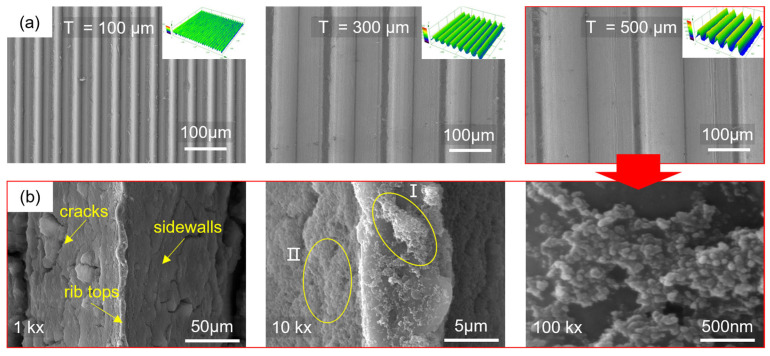
Surface morphology of microstructures: (**a**) SEM image and 3D profile of microarray channels; (**b**) nanostructure morphology after hydrophobic modification of *T* = 500 μm microstructures at different magnifications.

Figure 2 shows the three-dimensional morphology of the microstructures formed by the molding process and the surface nanostructures after hydrophobic modification. The microarray channel structure has clear 3D contours and is well-filled. Due to the rounded corners of the mold, the top of the microstructure is relatively flat. The surface of the microstructure with a diameter of 100 μm is rough and contains metal debris. It is also found that the number and size of the debris decrease as the microstructure scale increases. This is because the smaller-scale microarray channels are more densely arranged, and the friction conditions during the molding process are worse than those of large-scale microstructures, resulting in lower surface quality. For larger-scale microstructures, the microchannels are filled more fully. Figure 2b shows the surface morphology of the microstructure with a diameter of 500 μm after hydrophobic modification. The channel surface is covered with fluorinated silica, and cracks appear after drying. Cluster structures are observed in region I of the rib top, with an axial size of approximately 5 μm and a radial size of 1 to 3 μm. A layered structure is observed in region II of the channel sidewalls. Further observation revealed that the cluster structure is composed of small spheres with diameters ranging from 40 to 50 nm. The uneven distribution of the nanospheres increases the surface undulation of the cluster structure, and the uneven cluster structure can increase the surface roughness of the microstructure. This also demonstrates that the micro-nanostructures prepared through hot molding and surface modification have cross-scale characteristics, from nanoscale spherical structures to micron-scale cluster structures and then to hundred-micron-scale array channel structures. This multi-level composite micro-nanostructure will help improve surface hydrophobicity.

### 3.2. Condensation Behavior of Droplets on Microstructures

**Figure 3:**
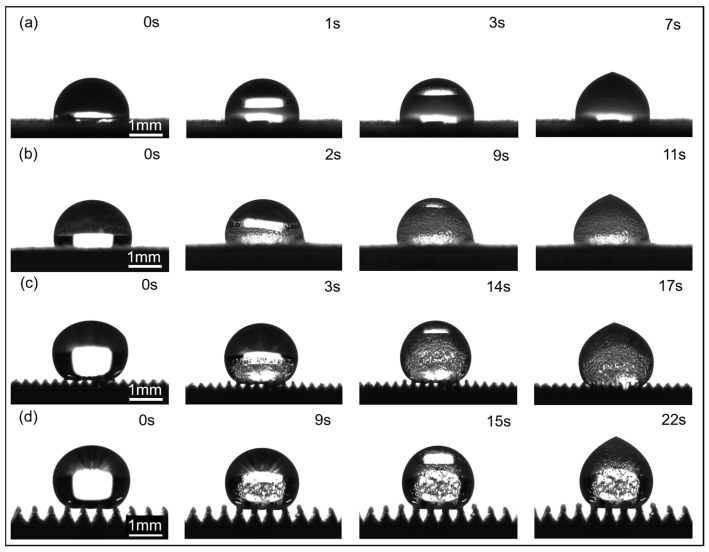
Freezing behavior of droplets on microstructures: (**a**) flat; (**b**) *T* = 100 μm; (**c**) *T* = 300 μm; (**d**) *T* = 500 μm.

To investigate the influence of surface microstructure size on condensation heat transfer property, single-droplet freezing experiments were conducted on supercooled microstructures, as shown in Figure 3. As can be seen, when the droplet first contacts the top of the microstructure, the *T* = 100 μm microstructure exhibits a Wenzel contact state, with the microstructure immersed in liquid. In contrast, the droplet on the *T* = 300 μm and *T* = 500 μm microstructures exhibits a Cassie contact state, with an air cushion forming between the droplet and the microstructure gap, maintaining a high contact angle. At −6 °C, the temperature drops from the bottom of the microstructure. Upon contact with the microstructure, the droplet rapidly condenses and nucleates, resulting in a distinct cold front and a transition from transparent to opaque. This is due to the transformation of the disordered state of water molecules into an ordered state of the ice lattice and the formation of cavities caused by air precipitation, which results in a change in the refractive index. For the *T* = 300 μm and *T* = 500 μm microstructures, the cold front initially freezes, moving upward in an arc-like pattern centered on the top of the microstructure and gradually merging into a flat surface. As the cold front gradually moves from the microstructure to the top of the droplet, the supercooled water inside the droplet transforms into solid ice crystals, eventually completely freezing the droplet. During this process, because the bottom of the droplet is already frozen onto the microstructure, forming a pinning, the liquid portion of the top free boundary is squeezed by the ice layer, causing the droplet’s top to freeze with a high curvature, forming a distinct white peak. Simultaneously, due to the sudden drop in temperature, a large number of bubbles are observed to precipitate within the droplet and become trapped within the ice layer. The presence of bubbles further exacerbates the uneven expansion and interfacial tension changes during freezing, increasing the local disturbances in ice crystal formation. Expansion cracks appear on the droplet surface due to volume expansion. Furthermore, in the Cassie state, the droplet rests on the air cushion at the top of the microstructure and between the channels. During freezing, the solid–liquid contact line is pinned by the microstructure. The formation of a solid–solid contact interface after freezing further stabilizes the contact between the droplet and the microstructure.

**Figure 4:**
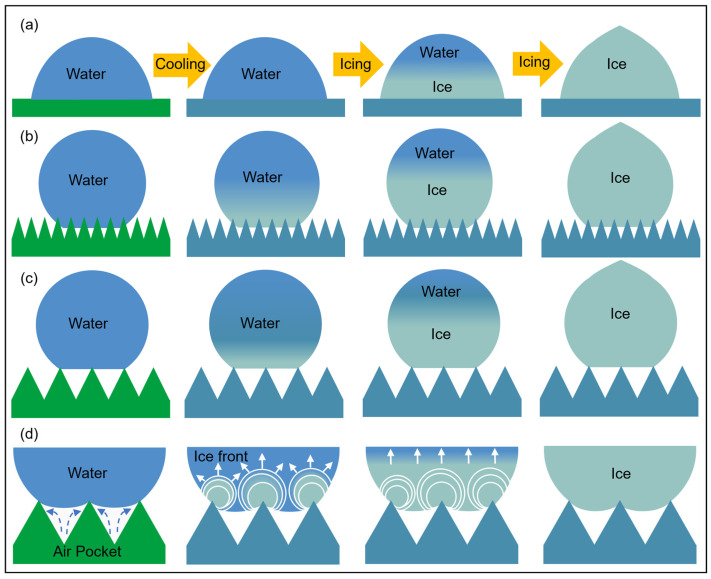
Schematic diagram of the droplet-freezing process: (**a**) flat; (**b**) smaller-scale microstructure; (**c**) larger-scale microstructure; (**d**) partial enlargement of (**c**), the blue dashed arrow indicates the suppression effect of air pockets on droplet wetting within the microstructure, while the white arrow indicates the direction of movement of the ice front during droplet freezing.

Figure 4 is a schematic diagram of the droplet freezing process on the flat, smaller-scale microstructure and the larger-scale microstructure. Figure 4d is a partial enlargement of Figure 4c. It can be seen that the contact state of droplets on the microstructure gradually transitions from the Wenzel state to the Cassie state as the microstructure size increases, and Figure 4d more intuitively reflects the inhibitory effect of air pockets in the microstructure on the wetting behavior of droplets. At the same time, it shows that compared with the flat microstructure, the microstructure can reduce nucleation sites and form pinning, causing the droplets to freeze at a high contact angle state.

The freezing of a droplet on the surface can be divided into two stages: ice crystal nucleation and crystal growth. In the first stage, the droplet maintains a high contact angle, allowing internal heat to dissipate through convection between the substrate and the air. The temperature drops until it reaches the saturated supercooling required for ice nucleation. At this point, the liquid water remains metastable, and ice crystals have not yet formed. The internal heat is dissipated through convection between the substrate and the air, and the temperature drops until it reaches the saturated supercooling state required for ice nucleation. At this time, the liquid water remains metastable, and ice crystals have not yet formed. When supercooling reaches the critical value, the top of the microstructure provides a nucleation site, lowering the nucleation barrier, and ice crystal nuclei are preferentially formed at the top of the microstructure. In the second stage, rapid kinetic crystal growth replaces the initial nucleation process. After the ice nucleus is formed, it grows in the height direction while diffusing radially. When the cold front reaches the apex of the droplet and the internal latent heat is completely released, the droplet enters a stable state.

It was also observed that the droplet freezing time increases with increasing microstructure size, and that droplets on all microstructures freeze longer than on flat substrates. A droplet on the *T* = 100 μm microstructure completely freezes in 11 s, a 57.1% improvement compared to a flat surface. A droplet on the *T* = 300 μm microstructure freezes completely in 17 s, while a droplet on the 500 μm microstructure freezes more slowly, slowing freezing to 22 s. These improvements represent 142.9% and 214.3% improvements, respectively.

**Figure 5:**
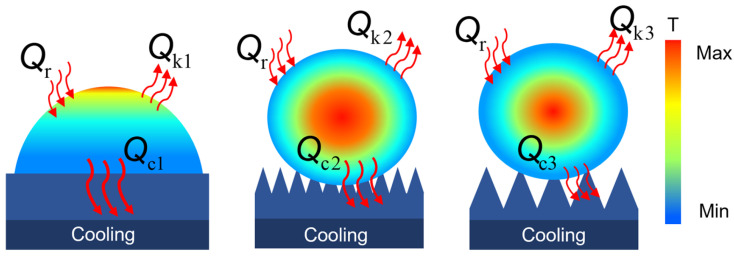
Temperature field distribution and heat loss model during water droplet freezing process.

This is because during the freezing process, the droplet loses heat from the gas–liquid and solid–liquid interface. Larger microstructures have a smaller contact area, resulting in less heat loss at the solid–liquid interface. Furthermore, the air cushion beneath the microstructures prevents further heat loss, delaying surface freezing, as shown in Figure 5. This process can be described by the following formula:

(1) $$∆Q=Q_{r}-(Q_{k}+Q_{c})$$

where ∆Q represents the rate of heat change of the water droplet on the superhydrophobic surface per unit time, $Q_{r}$ is the heat absorbed from the environment, and $Q_{k}$ and $Q_{c}$ correspond to the heat loss through the gas–liquid interface and solid–liquid interface, respectively.

**Figure 6:**
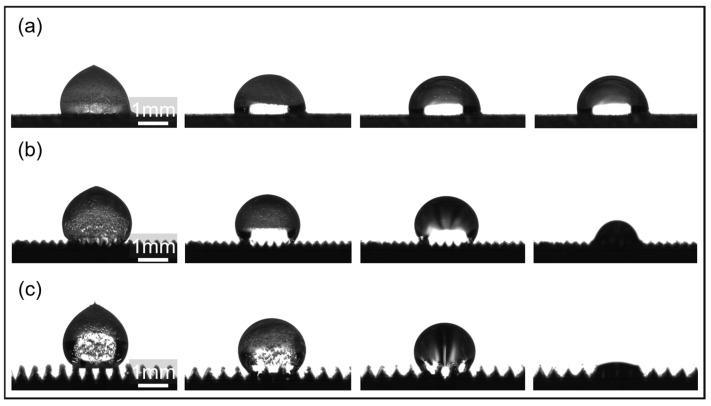
Melting process of droplets on microstructures: (**a**) *T* = 100 μm; (**b**) *T* = 300 μm; (**c**) *T* = 500 μm.

During melting, the ice crystals begin to melt from the bottom in contact with the microstructure, while the frost crust on the surface of the ice crystals also melts rapidly. The melted liquid portion flows along the channel under the suction of the microstructure, causing the ice crystals to fall further into the microstructure, accelerating the melting process, as shown in Figure 6. Due to insufficient suction capacity, the droplets on the *T* = 100 μm microstructure continue to remain hydrophobic after melting. The droplets in the *T* = 300 μm microstructure spread along the channel, but the droplets are pinned by the top of the microstructure and acted upon by surface tension. After stabilization, the bottom of the droplet forms an arc-shaped transition with the top of the microstructure. On the other hand, the droplets on the *T* = 500 μm microstructure continue to spread under the strong suction, and the liquid level eventually drops to slightly above the top of the microstructure.

As the microstructure substrate rapidly heats up, ice crystals at the microstructure interface melt into liquid. Simultaneously, the surface frost crust transforms into a thin liquid film through heat exchange with the air. When *T* = 100 μm, the capillary suction force of the microstructure is insufficient due to the dominant viscous resistance of the liquid. Melted droplets cannot spread, thus maintaining a hydrophobic state. The retained meltwater acts as an insulating layer, slowing heat exchange between the droplet and the microstructure and prolonging the melting time. When *T* = 300 μm, the capillary force provided by the microstructure balances the gravity of the droplet, forming a liquid bridge of constant curvature at the contact point between the droplet and the microstructure. When meltwater is drawn into the microstructure, it makes full contact with the channel sidewalls, enhancing heat transfer. Furthermore, the flowing liquid enters the microstructure, disrupting the air pocket and spreading along the channel, causing ice crystals to fall into the microstructure and accelerating the melting–suction process. When *T* = 500 μm, the channel aspect ratio increases, reducing the suction distance for the same volume of water and increasing the effective suction force of the microstructure. Once liquid water has completely entered the microstructure, it can be promptly transferred and removed, allowing the microstructure to restore its air pocket and maintain its good ice resistance the next time the droplet freezes. Therefore, the microstructure is the site of phase change, flow, and interfacial forces in the droplet.

### 3.3. Water Vapor Condensation Behavior on Microstructures

Single droplet freezing experiments have shown that hydrophobic microstructures exhibit significantly better freeze–thaw-pump property than hydrophilic surfaces. However, in practical applications, microstructured surfaces are often exposed to high-humidity and low-temperature environments, making it necessary to study the condensation behavior of water vapor on microstructures.

**Figure 7:**
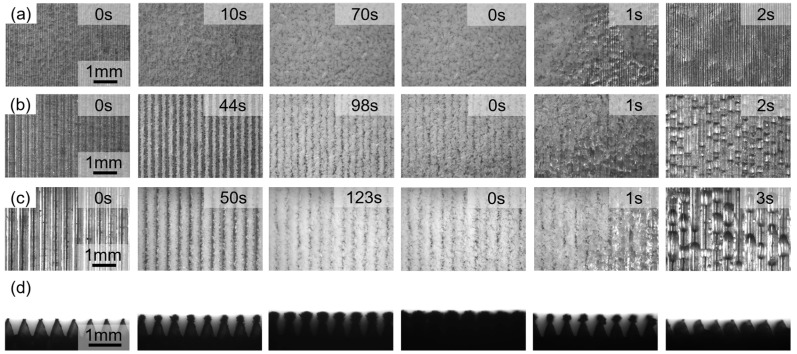
Frosting and defrosting process on microstructured surface: (**a**) *T* = 100 μm; (**b**) *T* = 300 μm; (**c**) *T* = 500 μm; (**d**) *T* = 500 μm parallel to the channel direction.

During the process of surface frost formation and icing, we found that ice crystals preferentially nucleated at the channel tops, as shown in Figure 7. Initially, ice crystals grew rapidly, continuing to grow until the entire surface was covered by ice. The duration of each stage increased with increasing channel size. When *T* = 100 μm, the ice layer rapidly grew and covered the rib tops within 10 s. By 70 s, the frost layer had completely covered the microstructure, with no apparent orientation in its distribution. When *T* = 300 μm, the ice layer growth slowed, completely covering the rib tops at 98 s. At this point, ice crystals were evenly distributed along the ribs and grew perpendicular to them. By 123 s, the frost layer had completely covered the channel gaps, but narrow gaps parallel to the ribs could still be observed between the frost layers. When *T* = 500 μm, the ice layer growth behavior was similar to that of 300 μm, but the freezing time was prolonged, and the gaps between the ice layers were reduced. Compared to the *T* = 100 μm microstructure, the frost layer spreading time for *T* = 300 μm and *T* = 500 μm microstructures increased by 40.0% and 75.7%, respectively.

On the surface of supercooled microstructures, the nucleation behavior of water vapor during condensation is significantly affected by the surface geometric characteristics. The local high curvature at the top of the microchannel and the extremely small curvature radius can effectively reduce the critical nucleation energy barrier, thereby promoting condensation nucleation. At the same time, water vapor is subject to geometric constraints when diffusing to the top of the channel, resulting in local enrichment in the sharp corner area, forming a higher water vapor concentration and making it easier to capture water vapor at the top of the microstructure. The supersaturation provides a stronger driving force for nucleation. In addition, the channel tip area can create more defects and active sites at the molecular scale, further serving as an efficient heterogeneous nucleation site. Therefore, under the synergistic effect of curvature effect, concentration enrichment, and heterogeneous nucleation sites, ice crystals preferentially nucleate at the top of the microstructure.

During the growth phase, the shallow channel depth and short heat conduction path from the substrate facilitate efficient heat exchange between water vapor and the top of the structure, resulting in a greater degree of supercooling and driving the nucleation and growth of ice crystals. Furthermore, the smaller channel size facilitates rapid diffusion of water vapor, ensuring timely replenishment of water vapor required for condensation, ultimately leading to rapid ice crystal coverage at the top. Observations along the channel indicate that ice crystals preferentially nucleate at the top of the microstructure. Their growth in the width direction is less constrained, allowing them to expand freely in three dimensions, forming a clustered structure. Within the channel, ice crystal growth is constrained by the sidewalls, extending primarily along the longitudinal direction of the channel. Furthermore, the rapid clustering of ice crystals at the top hinders further diffusion of water vapor into the channel, thereby suppressing the growth rate of ice crystals within the structure. For the *T* = 100 μm microstructure, when the growth length of ice crystals at the tops of adjacent ribs exceeds half the period, ice crystals bridge, and condensed water vapor subsequently aggregates primarily in the thickness direction, resulting in an overall non-oriented growth morphology of the ice layer. For the microstructure with *T* = 300 μm, the wider channel provides a diffusion channel for water vapor so that the slits parallel to the rib direction can be retained. When the channel period increases to *T* = 500 μm, the aspect ratio increases accordingly, and the water vapor diffusion efficiency is enhanced, thereby promoting the ice layer to fill the channel more fully, and the slit width also decreases accordingly. Therefore, the channel height determines the efficiency of the heat conduction path and water vapor diffusion and supply, affecting the coverage speed and growth morphology of ice crystals. The channel period determines whether ice crystal bridging behavior occurs or not and whether the final ice layer retains oriented slits or presents uniform filling characteristics. In addition, the three-dimensional free growth of ice crystals in the top area is in sharp contrast to the longitudinally restricted growth inside the channel, which is the result of the regulation of ice crystal morphology by channel geometric constraints.

During melting, the frost layer melts rapidly within 2–3 s. However, the high surface energy of the aluminum alloy with a microstructure of 100 μm prevents melted droplets from escaping the channel in time. Furthermore, due to the small periodicity of the microstructure, droplets coalesce and easily cross the channel, transforming into a continuous water film higher than the microstructure. Furthermore, it was observed that the water film takes 3 s to self-renew under the action of gravity alone. Although the water film can remove a large amount of water at a time, the renewal period is long. Furthermore, the thermal conductivity of water (0.55 W/mK) is much lower than that of aluminum (150 W/mK). The surface water film creates greater thermal resistance and is detrimental to heat transfer. At *T* = 300 μm, the increased channel width weakens the capillary capture ability of the microstructure. As the frost layer melts, it fails to form a liquid film and instead forms columnar droplets trapped on the channel sidewalls, with similar droplet volumes. At *T* = 500 μm, as the channel size increases further, the capillary force decreases again, and the melted droplets are elongated by gravity, extending the frost melting time to 3 s. At the same time, a large number of small droplets adhere to the side walls of the channel and the top of the microstructure, which is a mixed mode driven by surface free energy. The shape and size of the droplets show great inhomogeneity. This non-uniform distribution leads to fluctuations in local thermal resistance, thereby delaying the overall melting process. However, considering the water vapor condensation process, the *T* = 500 μm microstructure shows better anti-frost properties. Therefore, an efficient condensation surface that can increase the droplet nucleation density, reduce the droplet detachment size, and minimize the thermal barrier needs to simultaneously coordinate the inherent advantages of both droplet condensation and film condensation modes. The size-dependent phenomenon of ice crystal nucleation, growth, and icing on the triangular microchannel on the aluminum alloy surface is the joint result of the coupling effects involving microscale phase transition thermodynamics, interfacial energy effects, mass transfer/heat transfer limitations, and geometric constraints.

### 3.4. Freezing Behavior of Microstructured Surfaces After Hydrophobic Modification

A single micron-scale structure can prolong the freezing time of droplets and the frost formation time of the surface. By chemically modifying the microstructure surface with low surface energy through a nano-silica solution and constructing a hydrophobic micro-nano composite structure, the surface hydrophobicity can be further improved, and the anti-icing property of the microstructure can be significantly improved.

**Figure 8:**
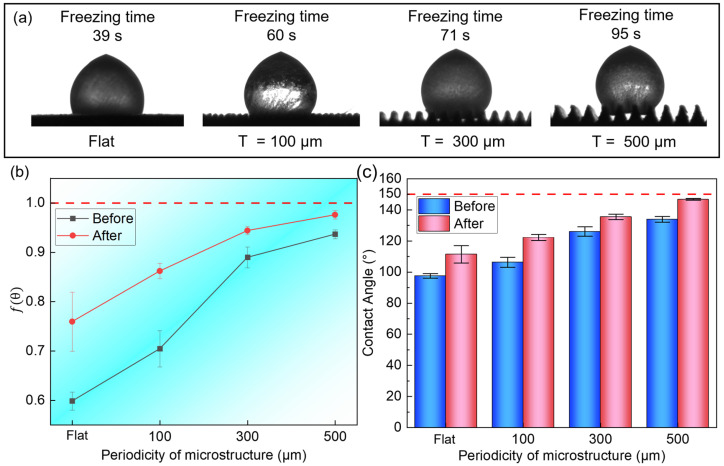
Freezing behavior of composite micro-nanostructure surfaces: (**a**) freezing time after modification; (**b**) binding geometry factor before and after modification; (**c**) water contact angle before and after modification.

As shown in Figure 8a, compared with the surface without hydrophobic treatment, the freezing time of droplets was significantly prolonged after chemical modification: the flat microstructure was delayed from 7 s to 39 s, a delay of 420%; the *T* = 100 μm microstructure was delayed from 11 s to 60 s, a delay of 445.5%; the *T* = 300 μm microstructure was delayed from 17 s to 71 s, a delay of 317.6%; and the *T* = 500 μm microstructure was delayed from 22 s to 95 s, a delay of 331.8%. In addition, compared with the initial plane, the freezing time of droplets on the surface of the micro-nano composite structure was delayed by up to 1166.67%, demonstrating excellent anti-icing performance. The freezing behavior of droplets is also different from that of single-channel structures. When frozen, the droplet first undergoes a stable period, and instantly becomes opaque as a whole before starting to freeze. The ice front gradually moves upwards from the contact point between the microstructure and the droplet until it is completely frozen.

**Figure 9:**
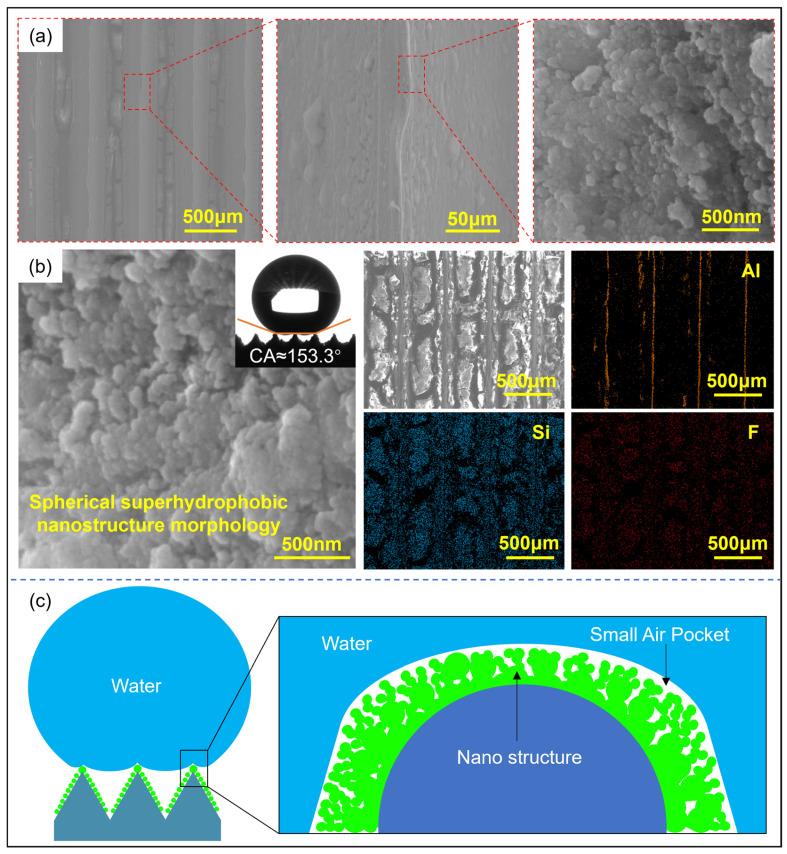
Surface morphology of *T* = 500 μm microchannel after chemical modification. (**a**) Micro-nanostructure; (**b**) contact angle and element distribution; (**c**) hydrophobic mechanism of the multi-level hierarchical structure.

The low-surface-energy fluorinated carbon chains in fluorosilane can provide a chemical basis for hydrophobicity. Simultaneously, the nanoscale cluster structure formed by the accumulation of silica nanoparticles on the micrometer-scale microstructure surface jointly constructs a rough micro-nano composite surface structure. These two effects synergistically create a superhydrophobic surface that prevents droplets from wetting into the microstructure interior. After the nanosphere structure covers the surface of the microstructure, a nano-scale concave–convex structure is formed, which increases the number of air pockets captured by the microstructure, reduces the solid–liquid contact area, and further improves the gas–liquid interface fraction, as shown in Figure 9c. Liu et al. [35] constructed a superhydrophobic surface (SHS) on AZ31 magnesium alloy using the electrodeposition method. The static contact angle reached 155°, and at −15 °C, the average ice nucleation time of 5 μL water droplets on the SHS surface was 14 min, which was delayed by 133.3% compared to the original surface (7 min). Progress analysis found that due to the micro/nano structure of superhydrophobic surfaces, the contact area with water droplets decreases. By reducing heat conduction, the formation of ice crystals is delayed, and this mechanism of delayed freezing is consistent with our findings.

At the same time, the triangular channel and the high curvature characteristics of the nanoparticles jointly pin the three-phase contact line, inhibiting the spreading of the droplets and significantly improving their hydrophobicity. This results in a maximum water contact angle of 153.3° on the composite surface. At the same time, the nanoscale structure can inhibit the nucleation of ice crystals [36] and increase the degree of supercooling. After the ice crystal nucleates, it quickly spreads to the entire droplet, driven by a larger degree of supercooling, and further triggers nucleation. The nucleation process needs to overcome the Gibbs free energy nucleation barrier, which can be described by the following formula [37]:

(2) $$∆G_{c}=∆G_{c}^{homo}\times f(m,x)$$

where f(m,x) is the geometric correction factor related to the contact angle θ, and 0 < $f\left(m,x\right)$ < 1, which can be simplified as follows:

(3) $$f\left(\theta \right)=\frac{\left(2+\cos\theta \right){\left(1-\cos\theta \right)}^{2}}{4}$$

The change in the nucleation barrier of the microstructure after hydrophobic modification can be calculated, as shown in Figure 8b. Therefore, the *f*(*θ*) on the modified surface increases compared to the unmodified microstructure surface, which means an increase in the nucleation barrier. At this time, the droplet must be supercooled more deeply, which requires a longer contact time to overcome the higher energy barrier to initiate ice crystal nucleation and then advance the ice front to completely freeze the droplet.

## 4. Conclusions

This study used a PVD-coated punch to form a microarray channel structure on an aluminum alloy through a hot-stamping process. The freezing behavior of droplets on the microarray channel and the frosting and defrosting process were analyzed. A superhydrophobic micro-nano composite structure was prepared through subsequent low-surface-energy fluoride modification. Droplet freezing delay experiments demonstrated that the micro-nano composite structure has excellent anti-icing properties. The main conclusions are as follows:

(1) The microarray channels formed by the hot molding process are fully filled and have clear outlines. A superhydrophobic micro-nano composite structure is prepared by spraying a fluorinated reagent. The surface of the structure has multi-scale characteristics due to the accumulation of nanospheres.
(2) The microstructures can store air and provide channels for water vapor diffusion, resulting in excellent anti-icing and anti-frost properties. As the structure size increases, the droplet freezing time and frost layer coverage time both increase. The freezing time of the *T* = 500 μm microstructure is 214.3% later than that of the flat sample (7 s → 23 s), and the frost layer coverage time is extended by 75.7% compared with the *T* = 100 μm microstructure (70 s → 123 s).
(3) The superhydrophobic micro-nano composite structure has excellent waterproof and anti-icing properties. The chemical basis of fluoride and the geometric constraints of the nanostructure show that its water contact angle reaches a maximum of 153.3°, and the droplet freezing time is delayed to 1166.67% of the initial plane (7 s → 95 s), indicating that the multi-level micro-nano composite structure can significantly improve the surface anti-icing property.

## Figures and Tables

**Table 1:** Comparison of microstructure anti-icing technology.

Microstructure	Substrate Material	Performance Testing Conditions (Water Droplet)	Core Index	Reference
Femtosecond laser	Silicone rubber	25 °C, ice wind tunnel	Dynamic anti-icing duration increased by 83.3% (72 s → 132 s)	[14]
Nanosecond laser + vacuum treatment	7075	atmospheric environment, 4–20 μL	Contact angle 173°; Excellent durability	[15]
Ultraviolet laser irradiation deposition	Pure copper	−17 °C, 10 μL	Freezing delay time is 3 times that of the original surface	[19]
Plasma etching	Silicon	−15 °C, 5 μL	Freezing time delayed to 1295 s	[23]
Electrochemical etching	Pure aluminum	−10 °C, 4 μL	Frost and melted water on the micro/nano structure surface can fall off more easily	[30]
High-speed wire electrical discharge machining	7075	Room temperature, 6 μL	Apparent contact angle 153°, contact angle hysteresis < 5°	[22]
Micro-milling + chemical modification	3A21 aluminum alloy	Room temperature, 4 μL	The contact angle is 156° Good corrosion resistance	[31]
Hot pressing + no oxide modification	6063	Room temperature, 5 μL	Liquid droplets are not easy to spread and easier to slide off	[32]

## Data Availability

The original contributions presented in this study are included in the article. Further inquiries can be directed to the corresponding authors.
